# Outer Membrane Vesicles and Soluble Factors Released by Probiotic *Escherichia coli* Nissle 1917 and Commensal ECOR63 Enhance Barrier Function by Regulating Expression of Tight Junction Proteins in Intestinal Epithelial Cells

**DOI:** 10.3389/fmicb.2016.01981

**Published:** 2016-12-15

**Authors:** Carina-Shianya Alvarez, Josefa Badia, Manel Bosch, Rosa Giménez, Laura Baldomà

**Affiliations:** ^1^Secció de Bioquímica i Biologia Molecular, Departament de Bioquímica i Fisiologia, Facultat de Farmàcia i Ciències de l’Alimentació, Universitat de BarcelonaBarcelona, Spain; ^2^Institut de Biomedicina de la Universitat de Barcelona, Institut de Recerca Sant Joan De DéuBarcelona, Spain; ^3^Unitat de Microscòpia Òptica Avançada, Centres Científics i Tecnològics, Universitat de BarcelonaBarcelona, Spain

**Keywords:** probiotics, gut microbes, *Escherichia coli*, phylogenetic group B2, membrane vesicles, tight junctions, intestinal barrier, TcpC

## Abstract

The gastrointestinal epithelial layer forms a physical and biochemical barrier that maintains the segregation between host and intestinal microbiota. The integrity of this barrier is critical in maintaining homeostasis in the body and its dysfunction is linked to a variety of illnesses, especially inflammatory bowel disease. Gut microbes, and particularly probiotic bacteria, modulate the barrier integrity by reducing gut permeability and reinforcing tight junctions. Probiotic *Escherichia coli* Nissle 1917 (EcN) is a good colonizer of the human gut with proven therapeutic efficacy in the remission of ulcerative colitis in humans. EcN positively modulates the intestinal epithelial barrier through upregulation and redistribution of the tight junction proteins ZO-1, ZO-2 and claudin-14. Upregulation of claudin-14 has been attributed to the secreted protein TcpC. Whether regulation of ZO-1 and ZO-2 is mediated by EcN secreted factors remains unknown. The aim of this study was to explore whether outer membrane vesicles (OMVs) released by EcN strengthen the epithelial barrier. This study includes other *E. coli* strains of human intestinal origin that contain the *tcpC* gene, such as ECOR63. Cell-free supernatants collected from the wild-type strains and from the derived *tcpC* mutants were fractionated into isolated OMVs and soluble secreted factors. The impact of these extracellular fractions on the epithelial barrier was evaluated by measuring transepithelial resistance and expression of several tight junction proteins in T-84 and Caco-2 polarized monolayers. Our results show that the strengthening activity of EcN and ECOR63 does not exclusively depend on TcpC. Both OMVs and soluble factors secreted by these strains promote upregulation of ZO-1 and claudin-14, and down-regulation of claudin-2. The OMVs-mediated effects are TcpC-independent. Soluble secreted TcpC contributes to the upregulation of ZO-1 and claudin-14, but this protein has no effect on the transcriptional regulation of claudin-2. Thus, in addition to OMVs and TcpC, other active factors released by these microbiota strains contribute to the reinforcement of the epithelial barrier.

## Introduction

The gastrointestinal epithelial layer is the first line of defense against pathogens and the surface where the host interacts with microbiota. This specialized epithelium forms a physical and biochemical barrier that maintains the segregation between host and intestinal microbiota. Several factors contribute to the epithelial barrier function, including the production of a mucin layer that covers the epithelial surface and prevents direct contact with intestinal microbes, the secretion of antimicrobial peptides, and the establishment of TJ between intestinal epithelial cells that seal the host tissue against the luminal environment. In addition, intestinal epithelial cells play a key role in sensing and integrating microbial signals that regulate the intestinal immune cell responses (reviewed by Turner, 2009; Wells et al., 2011; Peterson and Artis, 2014).

The TJ that connect adjacent intestinal epithelial cells are composed of different types of integral membrane proteins such as occludin, several claudins, tricellulin, and junctional adhesion molecules (Turner, 2009). Organization of the TJ structure depends on peripheral membrane proteins of the ZO family, ZO-1, ZO-2, and ZO-3, which bind to claudins and act as scaffolds anchoring the TJ transmembrane proteins to the actin cytoskeleton (Umeda et al., 2006; Shen et al., 2011). Claudins are a large family of TJ proteins that regulate paracellular permeability. To date, 27 claudin isoforms have been identified in humans. Some of them have a sealing function (like claudin-1), whereas others act as selective channels or pores for small charged molecules. Claudin-2, for instance, controls the movement of ions and enhances transepithelial water flux (Rosenthal et al., 2010).

The integrity of the epithelial barrier is critical in maintaining homeostasis in the body and its dysfunction is linked to inflammatory, allergic or metabolic diseases (Hering et al., 2012; Oshima and Miwa, 2016). Interaction between gut microbiota and the intestinal epithelium is crucial for the integrity of this barrier. Alterations in microbiota composition or aberrant responses to luminal bacteria or dietary components can result in increased intestinal permeability, which may lead to the development of such pathologies. In this context, many studies have been conducted to investigate the therapeutic potential of certain commensal and probiotic strains to ameliorate inflammatory bowel diseases in clinical trials (reviewed by Chibbar and Dieleman, 2015; Wasilewski et al., 2015) or in animal models of colitis (Ewaschuk et al., 2008; Arribas et al., 2009; Shen et al., 2012; Kang et al., 2013; Martin et al., 2014; Souza et al., 2016). In mice colitis models, beneficial bacteria reduce inflammatory cytokines, normalize gut permeability, and reinforce the epithelial barrier. Some studies indicate that these effects may be mediated, at least in part, by bacterial secreted factors (Ewaschuk et al., 2008; Martín et al., 2015) or by released membrane vesicles (Shen et al., 2012; Kang et al., 2013).

*Escherichia coli* Nissle 1917 (EcN) is a Gram-negative probiotic used to treat intestinal disorders, and particularly in maintaining remission of ulcerative colitis (Kruis et al., 2004; Chibbar and Dieleman, 2015). The strain, which was originally isolated from a soldier who survived a severe outbreak of diarrhea during the First World War, is a good colonizer of the human gut and positively affects gastrointestinal homeostasis and microbiota balance. It is well-known that EcN promotes anti-inflammatory modulation of the immune response (Trebichavsky et al., 2010). These effects have been mainly established from *in vitro* and *in vivo* experiments performed with live probiotic suspensions. We have recently shown that OMVs released by this probiotic can modulate the cytokine/chemokine response of gut epithelial and immune cells in *in vitro* and *ex vivo* cellular models (Fábrega et al., 2016). The probiotic EcN also positively modulates the intestinal epithelial barrier through both increased expression of secreted antimicrobial factors such as β-defensin-2 (Schlee et al., 2007; Fábrega et al., 2016) and upregulation and redistribution of the TJ proteins ZO-1 (Ukena et al., 2007), ZO-2 (Zyrek et al., 2007), and claudin-14 (Hering et al., 2014). Upregulation of claudin-14 in HT-29/B6 cells has been attributed to TcpC (Hering et al., 2014), an immunomodulatory protein secreted by uropathogenic *E. coli* strains that interferes with the host immune defense by inhibiting MyD88/Toll-like receptor 4 signaling cascade (Yadav et al., 2010; Snyder et al., 2013). However, the bacterial factors that regulate ZO-1 and ZO-2 expression have not been described to date.

Several facts indicate that the TJ strengthening capacity of EcN is mediated by factors released to the extracellular medium: (i) the TcpC activity on epithelial barrier function is associated with EcN culture supernatants (Hering et al., 2014), and (ii) EcN supernatants prevent barrier disruption by EPEC in polarized Caco-2 cell monolayers and confer protection against deleterious effects on paracellular permeability caused by virulence factors such as the serin protease Sat (secreted autotransporter toxin) (Toloza et al., 2015). The aim of this study was to define whether the TJ-barrier strengthening capacity of EcN is mediated by secreted factors or by OMVs using as a model T-84 and Caco-2 polarized monolayers. We extended the analysis to other *E. coli* strains of human intestinal origin from the reference collection (ECOR), and to the derived *tcpC* mutants. The activity of the two extracellular fractions (OMVs and soluble secreted factors) on the epithelial barrier function was evaluated by analyzing the expression and subcellular location of several TJ proteins. We show that both OMVs and soluble secreted factors from EcN and ECOR63 promote upregulation of ZO-1 and claudin-14, and down-regulation of claudin-2. The OMVs mediated effects are TcpC-independent.

## Materials and Methods

### Bacterial Strains and Growth Conditions

The probiotic strain EcN (serotype O6:K5:H1) was provided by Ardeypharm (GmbH, Herdecke, Germany). ECOR12 and ECOR63 are commensal *E. coli* strain isolated from healthy human stool samples (Ochman and Selander, 1984). ECOR57, from the same collection, was isolated from a healthy gorilla. EcN *tcpC::kan* and ECOR63 *tcpC::kan* were constructed in this work as described below. The laboratory *E. coli* strain HB101 (F^-^ *mcrB mrr hsdS20* (r_B_^-^ m_B_^-^) *recA13 leuB6 ara-14 proA2 lacY1 galK2 xyl-5 mtl-1*) was from the American Type Culture Collection (ATCC 33694). The *E. coli* strains S17 (λpir) (Biomedal) and EB6193 (RP4-2 *tet* Mu-1 kan::Tn*7* integrant; *leu-63*::IS*10 recA1 creC510 hsdR17 endA1 zbf-5 uidA*(MuI)::*pir*^+^*thi* Sp^r^/Sm^r^) (de la Riva et al., 2008) were used for cloning and propagation of the suicide plasmid pUT-miniTn5 Tc and derived recombinant constructs. Bacterial cells were grown at 37°C in Luria–Bertani broth (LB) or in DMEM (GIBCO) supplemented with 1% non-essential amino acids and 25 mM HEPES, with constant rotation (150 rpm). When required the following antibiotics were used at the indicated concentrations: ampicillin (Ap), 100 μg/ml; kanamycin (Km), 50 μg/ml; rifampicin (Rf), 50 μg/ml; and tetracycline (Tc), 30 μg/ml. Growth was monitored by measuring the optical density at 600 nm (OD_600_).

### Site Directed Mutagenesis of *tcpC* Gene

To construct *tcpC* knockout mutants, a 2059 pb-fragment encompassing the *tcpC* gene was amplified by PCR from EcN genomic DNA using oligonucleotides FWKO-tcpC CGCGGATCCGATGTCAGAAGCTTTACGAT and RVKO-tcpC CCGGAATTCCTTGCCCATGAAATAGATCT. Restriction sites for BamHI and EcoRI were incorporated at the 5′- ends of the primers (underlined) to facilitate directed cloning of the amplified fragment into plasmid pUC18Not (Biomedal). Disruption of the cloned *tcpC* gene was performed by insertion of the *kan* gene into the StuI site located 596 bp downstream of the ATG codon. The *kan* cassette was obtained by PCR amplification from plasmid pKD4 using primer Kn1 TCCCCCGGGGTGTAGGCTGGAGCTGCTTC and Kn2 TCCCCCGGGCATATGAATATCCTCCTTAG (Datsenko and Wanner, 2000), with the SmaI restriction site incorporated at their 5′-end (underlined). This process resulted in the recombinant plasmid pUC18NotI-*tcpC::kan*.

The *tcpC* knockout mutant derived from EcN was generated by antibiotic marker exchange using the suicide plasmid pUT-miniTn5 Tc (Biomedal) as described previously (Toloza et al., 2015). This vector contains the R6K origin of replication, which functions only in bacteria that contain the gene *pir*. The disrupted *tcpC::kan* gene was obtained by digestion of the recombinant pUC18NotI-*tcpC::kan* plasmid with NotI and further subcloned into the NotI restriction site of the suicide plasmid pUT-miniTn5 Tc. The resulting recombinant plasmid was propagated into strain EB6193 and introduced into *E. coli* S17 (λpir) by electroporation, to be used as a donor strain in mating experiments aimed at introducing the *tcpC* mutation into the EcN chromosome. A Rf-resistant derivative of EcN (Toloza et al., 2015) was used as the recipient. Transconjugants were selected for their resistance to Km and Rf on LB plates and further purified in this medium. After several rounds of growth at 30°C in this medium, colonies were screened for sensitivity to Ap and Tc in order to identify which transconjugants had undergone allelic exchange. Gene disruption of the target gene was verified by PCR (**Supplementary Figure S1**).

Since homologous recombination had not been successful with ECOR63, the *tcpC* mutant derived from this strain was constructed by the one-step inactivation method described elsewhere (Datsenko and Wanner, 2000). In this case, recombination requires the phage lambda Red recombinase, which is synthesized under the control of an inducible promoter on easily curable plasmid. We transformed ECOR63 with pKD46 harboring a heat-labile origin and the gene encoding the λRed recombinase under an arabinose-inducible promoter. To construct the ECOR63 *tcpC::kan* mutant, wild-type strain ECOR63 containing plasmid pKD46 was transformed with the *tcpC::kan* fragment obtained by PCR from the recombinant pUC18NotI-*tcpC::kan*, and grown in the presence of 100 mM arabinose at 30°C. Transformants were further grown at 37°C to loss the plamid. The ECOR63 *tcpC*::*kan* mutant was selected by screening for Km resistance and Ap sensitivity. The correct insertion of the mutation in the selected strain was confirmed by PCR (**Supplementary Figure S1**).

### Preparation of OMVs and Cell-Free Supernatant Fractions

Cell free supernatants (CF-SN) were obtained from bacterial cultures in LB medium or DMEM supplemented with 1% non-essential aminoacids and 25 mM HEPES as described previously (Toloza et al., 2015). Briefly, bacterial cells were pelleted by centrifugation at 10,000 × *g* for 20 min at 4°C; the supernatants were filtered through a 0.22 μm-pore-size filter (Merck Millipore) to remove residual bacteria and concentrated using a Centricon^®^ Plus-70 filter device with a molecular weight cutoff of 10 KDa (Merck Millipore). OMVs were isolated from this CF-SN fraction by ultracentrifugation at 150,000 × *g* for 1 h at 4°C in an Optima^TM^ L-90K ultracentrifuge (Beckman Coulter) as described before (Aguilera et al., 2014). The supernatant obtained in this step was concentrated twice and used as COF-SN fraction. Pelleted OMVs were washed twice, resuspended in an adequate volume of phosphate buffered saline (PBS) and stored at -20°C. Protein concentration was determined by the method of Lowry et al. (1951).

### Cell Culture and Growth Conditions

The human colonic cells lines Caco-2 (ATCC HTB37) and T-84 (CCL-248) were obtained from the American Type Culture Collection. The culture medium for Caco-2 cells was DMEM High Glucose supplemented with 10% (v/v) FCS, whereas for T-84 cells the medium was DMEM/F12 Glutamax medium (Gibco-BRL) with 5% (v/v) FCS. In both cases, media contained 25 mM HEPES, 1% non-essential amino acids, penicillin (100 U/ml) and streptomycin (100 μg/ml) (Gibco-BRL). Cultures were incubated at 37°C with 5% CO_2_. Cells were routinely subcultured once a week with trypsin-EDTA (Gibco-BRL) and seeded at a density of 2 × 10^5^ cells in 55 cm^2^ dishes (Caco-2) or in 75 cm^2^ flasks (T-84) for propagation.

### Transepithelial Resistance Measurement

T-84 cells (1 × 10^5^ cells/cm^2^) were seeded on the apical compartment of 12 mm polycarbonate Transwell cell culture inserts (0.4 μm, Transwell Millipore). The basolateral compartment contained 1.5 ml of the culture medium supplemented with 10% (v/v) FCS, penicillin and streptomycin. Cells were grown during 9 days. During growth and differentiation the medium was changed every 2 days in both compartments. Monolayer integrity was controlled by measurement of the transepithelial electrical resistance (TER) and by visual assessment of cell layer integrity under the microscope. Prior to apical stimulation with OMVs or culture supernatants (CF-SN or COF-SN), the medium was changed to DMEM/F12 Glutamax containing 25 mM HEPES, 1% non-essential amino acids, and gentamicin (100 μg/ml).

TER was measured with a Millicel-ERS-2 volt-ohmmeter (Millipore). Before measurement, electrodes were equilibrated and sterilized according to the manufacturer’s recommendations. The ohmic resistance of a blank (culture insert without cells) was measured in parallel. To obtain the sample resistance, the blank value was subtracted from the total resistance of the sample. The final unit area resistance (Ω.cm^2^) was calculated by multiplying the sample resistance (Ω) by the effective area of the membrane (1.12 cm^2^).

Stimulations were performed at initial TER values greater than 1000 Ω.cm^2^. CF-SN (2 mg/ml), COF-SN (0.5 mg/ml), or OMVs (0.1 mg/ml) were added to the apical compartment. After incubation for 24 h at 37°C, T-84 monolayers cells were washed with PBS. TER was measured after 1 h stabilization in the presence of fresh medium without FCS and antibiotics.

### RNA Isolation and Quantitative Reverse Transcription PCR (RT-qPCR)

T-84 (1 × 10^5^ cells/cm^2^) and Caco-2 (0.5 × 10^5^ cells/cm^2^) were seeded on 12-well plates and cultured for 9 days. After 4 h stimulation with COF-SN (0.5 mg/ml) or OMVs (0.1 mg/ml), total RNA was extracted from epithelial cells by using the Illustra RNAspin Mini kit (GE Healthcare) according to the manufacturer’s instructions. The concentration and purity of RNA samples were assessed by the ratio of the absorbance at 260 and 280 nm in a NanoDrop^®^ spectrophotometer. RNA integrity was verified by visualization of 28S and 18S rRNAs after 1% agarose/formaldehyde gel electrophoresis.

RNA (1 μg) was reverse transcribed using the High Capacity cDNA Reverse Transcription kit (Applied Biosystems) in a final volume of 20 μl following manufacturer’s recommendations. RT-qPCR reactions were performed in a StepOne Plus PCR cycler (Applied Biosystems) by using the Taqman Gene Expression Master Mix, and the Taqman probes and primers for human ZO-1, ZO-2, and occludin (Applied Biosystems), or SYBR^®^ Green PCR master mix (Applied Biosystems) and specific oligonucleotides for claudin-1 (Satake et al., 2008), claudin-2 (Zhang et al., 2013), and claudin-14 (BIORAD).

The standard PCR program used was: one denaturation cycle for 10 min at 95°C followed by 40 cycles of 15 s at 95°C and 1 min at 60°C. A control reaction was performed in the absence of RNA. The housekeeping gene β-actin was used as a normalizing gene. Relative gene expression was calculated as fold-change compared with control and calculated by means of ΔΔCt formula.

### Immunoblotting of TJ Proteins

T-84 cells were grown for 9 days as indicated above for RNA isolation. After 24 h stimulation with COF-SN or OMVs, cells were rinsed in PBS and incubated with lysis buffer (50 mM HEPES pH 7.4, 1% Triton X-100, 0.2% sodium deoxycholate, 0.1% SDS, 150 mM NaCl, 1.5 mM MgCl_2_, 1 mM EGTA, containing 20 μg/ml protease inhibitor cocktail) at 4°C for 1 h. Then, cells were scraped off with a rubber policeman, transferred to a chilled Eppendorf tube and homogenized for 40 s whilst on ice using an electrical homogenizer. Samples were then centrifuged at 20,800 × *g* for 15 min at 4°C, and the supernatant was collected and kept at -80°C until use.

Proteins were separated on 10% SDS-PAGE, and transferred to a Hybond-P polyvinylidene difluoride membrane by using a Bio-Rad MiniTransblot apparatus. For ZO-1 analysis 7% SDS-PAGE gels were used. The membrane was blocked in PBS-0.05% Tween-20 and 5% skimmed milk (blocking solution) for 1 h at room temperature, and then incubated with specific antibodies against ZO-1 (1:100 dilution), claudin-2 (1:1,000 dilution), occludin (1:1000 dilution) or β-actin (Sigma 1:10,000 dilution) for 16 h at 4°C. The secondary antibody was donkey anti-rabbit IgG conjugated to horseradish peroxidase, diluted 1:10,000 in blocking solution. β-Actin served as a loading control to normalize protein expression levels. The protein-antibody complex was visualized by using the ECL Plus Western blotting detection system (Amersham Pharmacia Biotech). For protein quantification, densitometry analysis was done with the software package Image Studio^TM^ Lite from LI-COR Biosciences.

### Immunofluorescence Labeling

Caco-2 cells were grown for 5–7 days in an 8-well chamber slide (μ-Slide 8 well Glass bottom, Ibidi). Then, cells were stimulated with COF-SN (0.5 mg/ml) or OMVs (0.1 mg/ml) for 24 h at 37°C. After washing with PBS cells were fixed with 3% paraformaldehyde in PBS, permeabilized with 0.05% saponin (Sigma–Aldrich) and blocked using PBS containing 1% bovine serum albumin. The TJ proteins ZO-1, claudin-2 and occludin were stained using, respectively, anti-ZO-1 (5 μg/ml, Invitrogen), anti-claudin-2 (5 μg/ml, Abcam) rabbit IgG antibodies, and anti-occludin mouse IgG antibody (0.5 μg/ml, Invitrogen) for 16 h at 4°C, followed by incubation with Alexa Fluor 488-conjugated F(ab’)2 goat anti-rabbit IgG (H+L) (5 μg/ml, Invitrogen) or Alexa Fluor 488 - conjugated F(ab’)2 goat anti-mouse IgG (H+L) (5 μg/ml, Invitrogen) for 2 h at 37°C. Nuclei were labeled with DAPI (0,125 μg/ml, Sigma–Aldrich) for 20 min at room temperature.

### Confocal Microscopy and Image Analysis

Confocal microscopy was carried out using a Leica TCS SP2 confocal microscope equipped with an Ar, an Ar-UV and a HeNe lasers. 12-bit images were obtained using a 63× 1.32NA oil immersion objective and an image resolution of 0.232 × 0.232 × 0.488 μm/voxel (*x*, *y*, *z*, respectively). Images were analyzed using Fiji (Schindelin et al., 2012). For quantitative analysis of ZO-1 and occludin, TJ were traced using the Tubeness plugin and projected by maximum intensity projection. The projected image was segmented using Li’s Minimum Cross Entropy thresholding method (Li and Tam, 1998) and the resulting segmented region of interest was used to measure mean intensity in the maximum intensity projected original image (**Supplementary Figure S2**).

### Statistical Analysis

Statistical analysis was performed using SPSS (version 20.0, Chicago, IL, USA) software package with pooled data from at least three independent biological replicates. The values for all measurements are presented as the mean ± standard error. Differences between more than two groups were assessed using one-way ANOVA followed by Tukey’s test. Significance was stablished when *p* ≤ 0.05.

## Results

### ECOR Strains That Contain the *tcpC* Gene Increase TER in T-84 Monolayers

The positive effects of the probiotic EcN on intestinal barrier function have been attributed to TcpC, a protein that mediates upregulation of the TJ protein claudin-14 (Hering et al., 2014). To search for other intestinal *E. coli* strains with TJ-barrier-strengthening ability, we screened the ECOR collection (Ochman and Selander, 1984) for the presence of *tcpC* sequences. Using the information available at NCBI^1^ we identified five *tcpC*-positive ECOR strains, namely ECOR53, 56, 57, 60, and 63 (Schubert et al., 2010). Like EcN, all of the strains belong to the phylogenetic group B2, associated with virulent strains that cause extra-intestinal infections. ECOR53 and ECOR63 were isolated from healthy human stools, whereas ECOR56 and ECOR60 were isolated from patients with urinary tract infection. ECOR57 was from a healthy gorilla (STEC Center)^2^. Studies to identify potential virulence markers among the ECOR strains showed that all of these strains, except ECOR57 and ECOR63, expressed functional haemolysin and caused strong cytotoxicity in the murine macrophage cell line J774 (Lai et al., 1999). With this information, we excluded the cytotoxic strains from this study.

**FIGURE 1:**
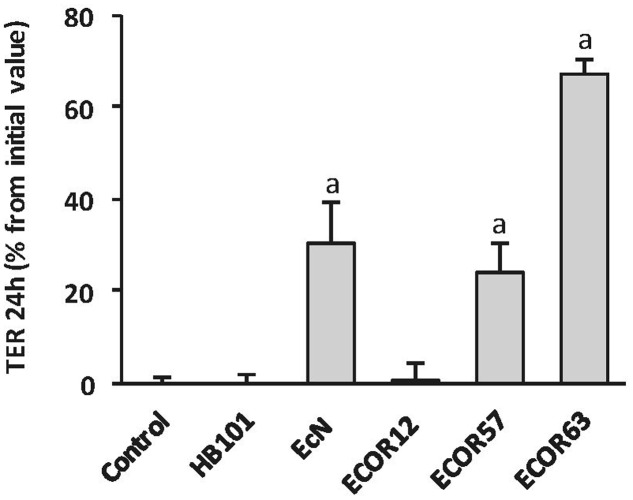
***Escherichia coli* reference collection (ECOR) strains bearing *tcpC* gene increase TER in T-84 cell monolayers.** The effect on TER of the indicated *Escherichia coli* strains from the ECOR collection was analyzed in T-84 monolayers in comparison with the probiotic EcN and the laboratory strain HB101. TER values were measured before and after 24 h incubation with cell-free supernatants (CF-SN) (2 mg protein/ml) collected from LB cultures (*n* = 3 independent biological replicates). Data are presented as percentage of increase in TER from the initial value. ^a^significance against untreated control cells (*p* ≤ 0.001).

We previously showed that EcN CF-SN have a positive impact on the TER of epithelial cell monolayers and prevent barrier disruption caused by pathogens (Toloza et al., 2015). To test the ability of *tcpC*-positive strains ECOR63 and ECOR57 to strength the epithelial barrier, T-84 monolayers were incubated for 24 h with CF-SN (2 mg/ml) collected from LB cultures, and TER was measured before and after the treatment period. CF-SN obtained from EcN cultures (positive control) or from HB101 (negative control) were processed in parallel. We also included the intestinal isolate ECOR12 (*tcpC-*negative) for comparison. Only the *tcpC*-positive strains increased TER, whereas the laboratory strain HB101 or the commensal ECOR12 had no effect on this parameter (**Figure 1**). These results are in accordance with those reported by Hering et al. (2014). As ECOR57 is not of human origin we selected ECOR63 for further studies.

### The Positive Effects of EcN and ECOR63 on TER Are Mediated by Soluble and Vesicle-Associated Factors

**FIGURE 2:**
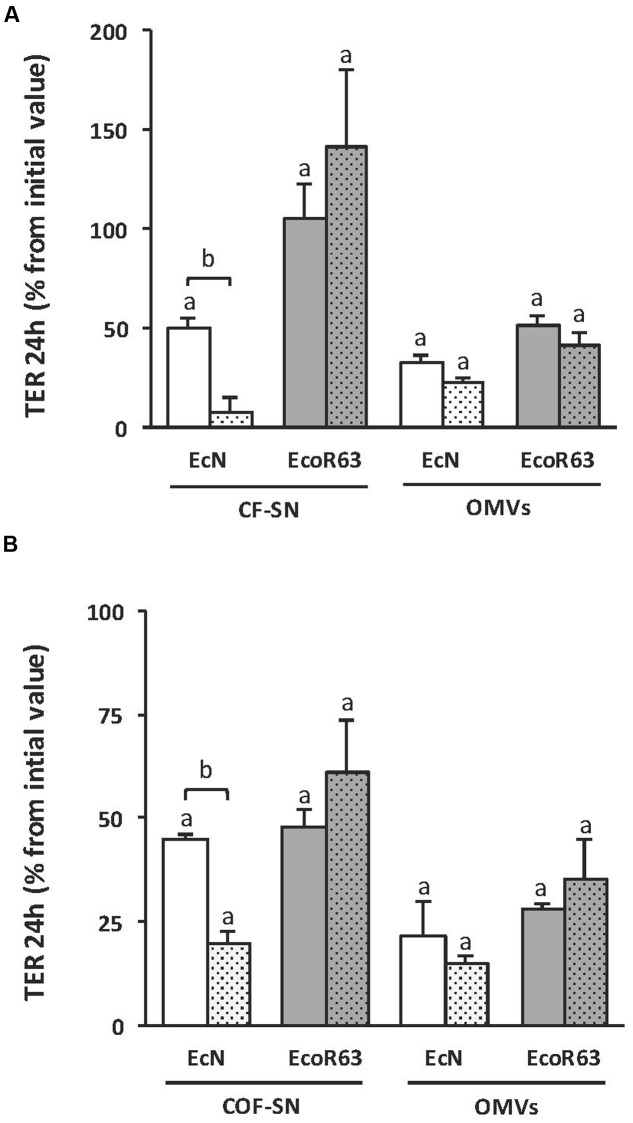
**The impact of ECOR63 and EcN on TER depends on both released soluble factors and OMVs.** **(A)** TER analysis of T-84 monolayers after 24 h incubation with CF-SN (2 mg/ml) (containing both released soluble mediators and OMVs) or isolated OMVs (0.1 mg/ml) from overnight cultures in LB medium of the wild-type strains EcN (white bars) and ECOR63 (gray bars) and from their corresponding *tcpC* mutants (doted bars). **(B)** TER analysis of T-84 monolayers after 24 h incubation with COF-SN (0.5 mg/ml) (containing only released soluble mediators) or isolated OMVs (0.1 mg/ml) from DMEM exponential cultures of the same wild-type strains (plain bars) and *tcpC* mutants (doted bars). In both panels, data are presented as changes in TER (%) from the initial value (*n* = 3 independent biological replicates). ^a^significance against untreated control cells (*p* ≤ 0.05); ^b^significance *tcpC* mutant vs. wild-type (*p* ≤ 0.002).

Since no information was available on the mechanism of TcpC secretion, we sought to analyze whether the TcpC effect was associated with OMVs. For this approach, we constructed *tcpC* mutants derived from both EcN and ECOR63 strains. These mutants, as well as the parental wild-type strains, were grown overnight in LB. Cultures were centrifuged and the supernatant was filtered to eliminate bacteria and obtain the corresponding CF-SN samples. An aliquot of each CF-SN was centrifuged at 150,000 × *g* for 1 h to isolate OMVs. The potential of these fractions to stimulate TER was analyzed on T-84 cell monolayers after 24 h incubation (**Figure 2A**). Regarding the probiotic EcN, the *tcpC* mutation significantly diminished the capacity of CF-SN to increase TER values. In contrast, CF-SN collected from the ECOR63 *tcpC* mutant increased TER to levels comparable to the wild-type strain. Analysis of TER in monolayers challenged with bacterial OMVs (0.1 mg/ml) revealed the potential of EcN and ECOR63 vesicles to increase TER. For both strains, this effect was TcpC- independent.

Protein components of the rich LB medium interfere with the protein quantification of CF-SN samples. Therefore, to better analyze the effect of released soluble mediators on TER, wild-type EcN and ECOR63, and the derived *tcpC* mutants were grown in DMEM. After 8 h growth (exponential phase), cells were removed by centrifugation and the CF-SN were processed to isolate OMVs from the secreted soluble fraction (COF-SN). The potential of these fractions to stimulate TER was analyzed on T-84 cell monolayers after 24 h incubation (**Figure 2B**). The results obtained after stimulation with OMVs (0.1 mg/ml) or COF-SN (0.5 mg/ml) collected from DMEM cultures were comparable to those achieved with samples isolated from LB cultures. Introduction of the *tcpC* mutation only significantly diminished the barrier strengthening capacity of COF-SN from EcN. Overall, these results indicate that both soluble and vesicle-associated factors mediate the positive effects of EcN and ECOR63 on TER. Moreover, they suggest that TcpC secretion is not associated with OMVs, and that, in addition to TcpC, commensal ECOR63 releases other factors that can modulate the strength of the epithelial barrier.

### Expression Analysis of TJ Proteins in Intestinal Epithelial Cells Challenged with OMVs or Soluble Factors Released by EcN and ECOR63

**FIGURE 3:**
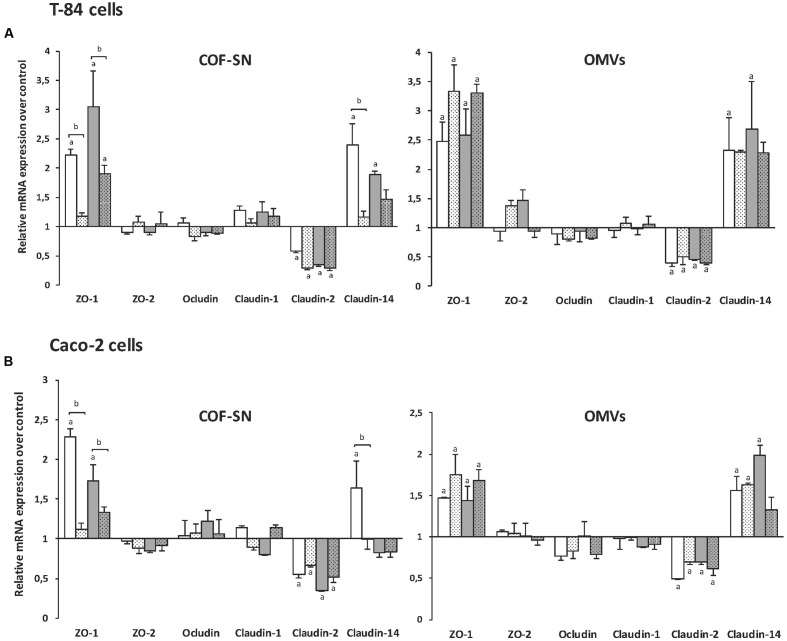
**Gene expression levels of tight junction proteins in the intestinal epithelial cell lines T-84 **(A)**** or Caco-2 **(B)**. Cell monolayers were challenged for 4 h with COF-SN (0.5 mg/ml) or OMVs (0.1 mg/ml) from EcN (white bars), EcN *tcpC::kan* (doted white bars), ECOR63 (gray bars) or ECOR63 *tcpC::kan* (doted gray bars). Relative mRNA levels of the indicated proteins were measured by RT-qPCR, using β-actin as the reference gene. Data are presented as fold-change compared to untreated control cells (*n* = 3 independent biological replicates). ^a^Significance against untreated control cells (*p* ≤ 0.04); ^b^significance *tcpC* mutant vs. wild-type (*p* ≤ 0.02).

As the observed effects on TER pointed to changes in TJ proteins, expression of genes encoding several barrier-relevant TJ proteins was analyzed in T-84 cells. In this study, we included proteins known to be regulated by the probiotic EcN, such as ZO-1, ZO-2, and claudin-14 (Ukena et al., 2007; Zyrek et al., 2007; Hering et al., 2014), and others like occludin, claudin-1 and claudin-2. T-84 monolayers (9 days post-confluence) were apically stimulated for 4 h with OMVs (0.1 mg/ml) or COF-SN (0.5 mg/ml) collected from exponential DMEM cultures of the two wild-type strains EcN and ECOR63, and from the *tcpC* mutants. Untreated monolayers served as a control. The mRNA levels of the indicated TJ proteins were measured by RT-qPCR (**Figure 3A**). The results show transcriptional regulation of ZO-1, claudin-2, and claudin-14 by the two types of bacterial samples (OMVs and COF-SN) from either EcN or ECOR63. Specifically they promoted upregulation of ZO-1 and claudin-14, and downregulation of claudin-2 (**Figure 3A**, plain bars). In contrast, mRNA levels of ZO-2, occludin and claudin-1 were not significantly altered under any incubation condition, as their mRNA levels remained similar to those of control monolayers.

Regarding the effect of COF-SN, upregulation of ZO-1 and claudin-14 was almost abolished by TcpC deficiency in EcN, and significantly weakened in the case of ECOR63 *tcpC* (**Figure 3A**, dotted bars). These results show that TcpC contributes to the transcriptional regulation of ZO-1 and claudin-14, and suggest that other released factors may contribute to this regulation in ECOR63. In contrast, TcpC deficiency did not result in noticeable changes in claudin-2 mRNA levels compared to COF-SN collected from wild-type EcN and ECOR63, thus ruling out the contribution of TcpC to the transcriptional control of claudin-2. Considering the effect of OMVs, no significant differences in the mRNA levels of ZO-1, claudin-2 or claudin-14 were observed between cells stimulated with OMV samples isolated either from the wild-type or the *tcpC* mutants (**Figure 3A**).

The expression of TJ proteins was also analyzed in Caco-2 monolayers. In general, the results show a similar transcriptional pattern, that is, upregulation of ZO-1 and claudin-14, and down-regulation of claudin-2 (**Figure 3B**). As in T-84 cells, transcriptional regulation of these proteins by OMVs was not affected by the *tcpC* mutation, but the relative mRNA levels of ZO-1 and claudin-14 were lower in Caco-2 cells (below 2-fold). Regarding stimulations with COF-SN, expression of ZO-1 and claudin-2 followed the same pattern as in T-84 cells. It was noticeable that the effect of COF-SN from ECOR63 and ECOR63 *tcpC* on ZO-1 expression was clearly lower than in the T-84 cell line, with relative mRNA levels that were around 50% of those achieved in T-84 monolayers (**Figure 3**). The lower response of Caco-2 cells to ECOR63 COF-SN could explain the lack of claudin-14 induction in this cell line. A 50% reduction in the relative claudin-14 mRNA levels observed in T-84 cells challenged with ECOR63 COF-SN (**Figure 3A**) may correlate in Caco-2 cells with mRNA levels close to those of untreated cells (**Figure 3B**).

**FIGURE 4:**
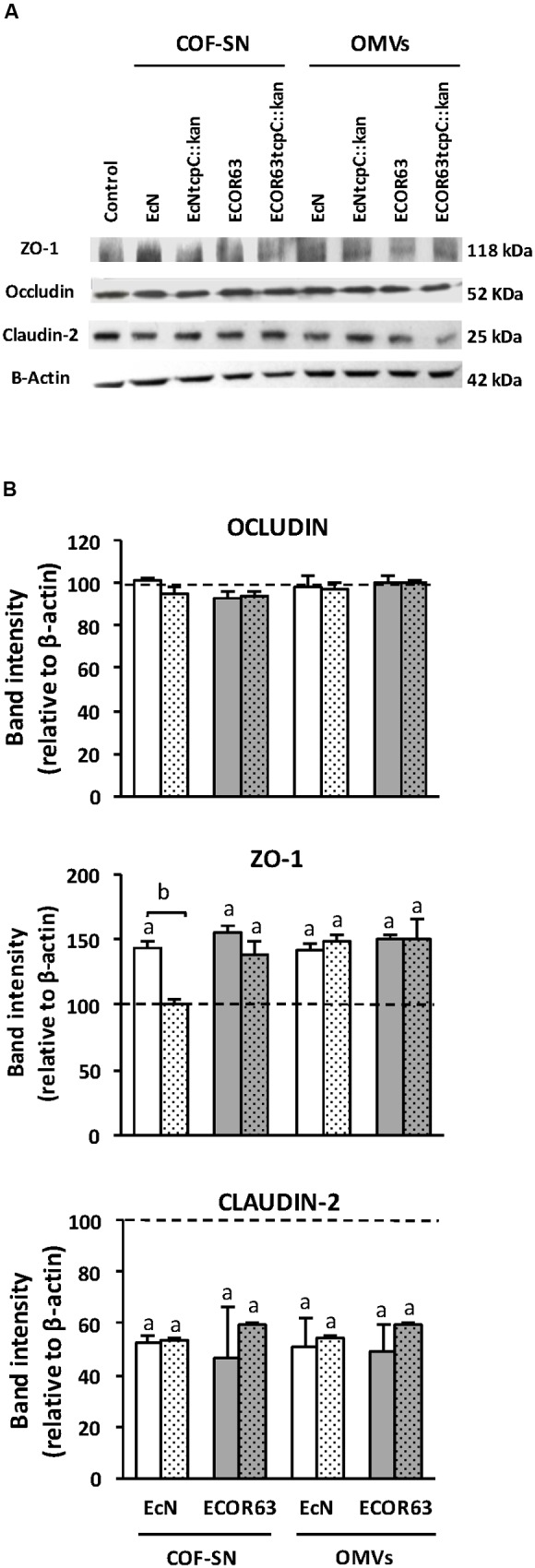
**Western blot analysis of TJ proteins in T-84 cell monolayers treated with COF-SN or OMVs from the indicated bacterial strains.** Cell monolayers were challenged for 24 h with COF-SN (0.5 mg/ml) orOMVs (0.1 mg/ml) from EcN (white bars), EcN *tcpC::kan* (doted white bars), ECOR63 (gray bars) or ECOR63 *tcpC::kan* (doted gray bars). Occludin, ZO-1 and claudin-2 were immunodetected with specific antibodies. **(A)** Representative Western blots of three independent experiments areshown. **(B)** Densitometric quantification of the TJ proteins (*n* = 3 independent biological replicates). Values were normalized to λ-actin. Normalized values from untreated control cells were set as 100% and indicated by a dashedline. ^a^Significance against untreated control cells (*p* ≤ 0.04); ^b^significance *tcpC* mutant vs. wild-type (*p* = 0.001).

We next undertook Western blot experiments to assess the regulation of ZO-1 and claudin-2 expression by soluble and vesicle-associated factors secreted by EcN and ECOR63. Occludin expression was analyzed as a control of a non-regulated protein. Immunoblotting analysis was performed in T-84 cell monolayers after 24 h incubation with extracellular samples from both wild-type and *tcpC* mutant strains (**Figure 4**). Densitometry quantification and normalization to β-actin confirmed downregulation of claudin-2 by all samples, with a 30–40% reduction in claudin-2 levels compared to the untreated control (**Figure 4B**). Regarding ZO-1, the results depend on whether T-84 cells were incubated with COF-SN or OMVs. Significant increases in ZO-1 were observed in cells treated with either wild-type EcN or ECOR63 COF-SNs. As expected, in cells incubated with COF-SN from the EcN *tcpC* mutant, ZO-1 protein levels did not significantly differ from those of untreated control cells. However, although ZO-1 levels achieved upon stimulation with supernatants from the ECOR63 *tcpC* mutant were slightly lower, they did not significantly differ from those of cells incubated with supernatants from the wild-type strain. Consistent with RT-qPCR data, OMVs triggered a significant increase in ZO-1 protein levels independently of the producer strain (**Figure 4B**). Quantification of occludin levels also confirmed that expression of this protein remained similar to those of untreated monolayers.

### Immunofluorescence Microscopy Analysis of TJ Confirm Regulation of ZO-1 and Claudin-2 by Factors Secreted by EcN and ECOR63

**FIGURE 5:**
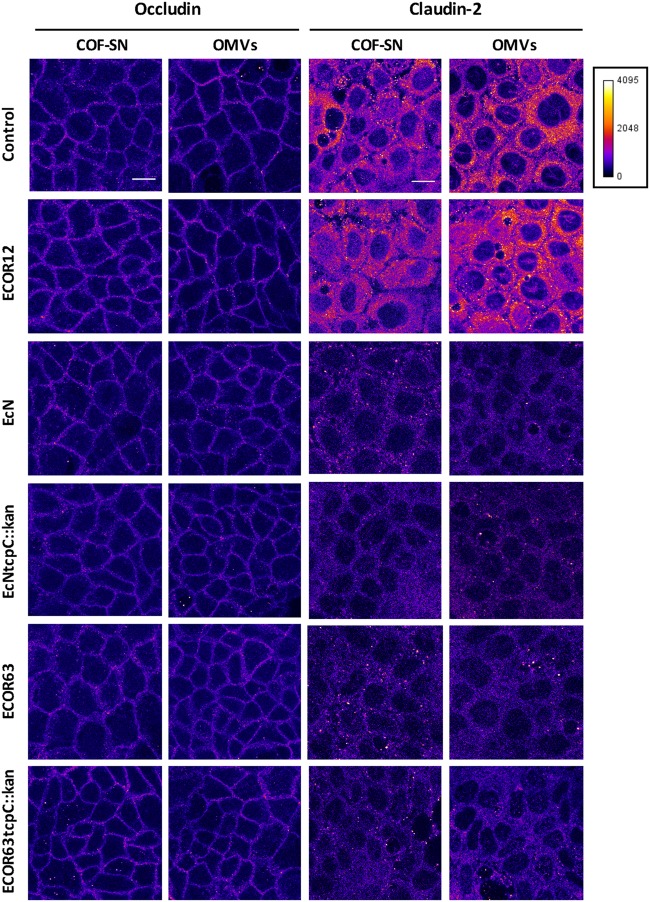
**Immunofluorescence staining for occludin and claudin-2 in Caco-2 cells treated for 24 h with COF-SN or OMVs from the indicated bacterial strains.** Immunostaining of occludin **(left)** and claudin-2 **(right)** was carried out in Caco-2 cells at 5 days after seeding (*n* = 3 independent biological replicates). Images are color coded with Fire look-up table and its calibration bar is shown on the right. Scale bar, 20 μm.

**FIGURE 6:**
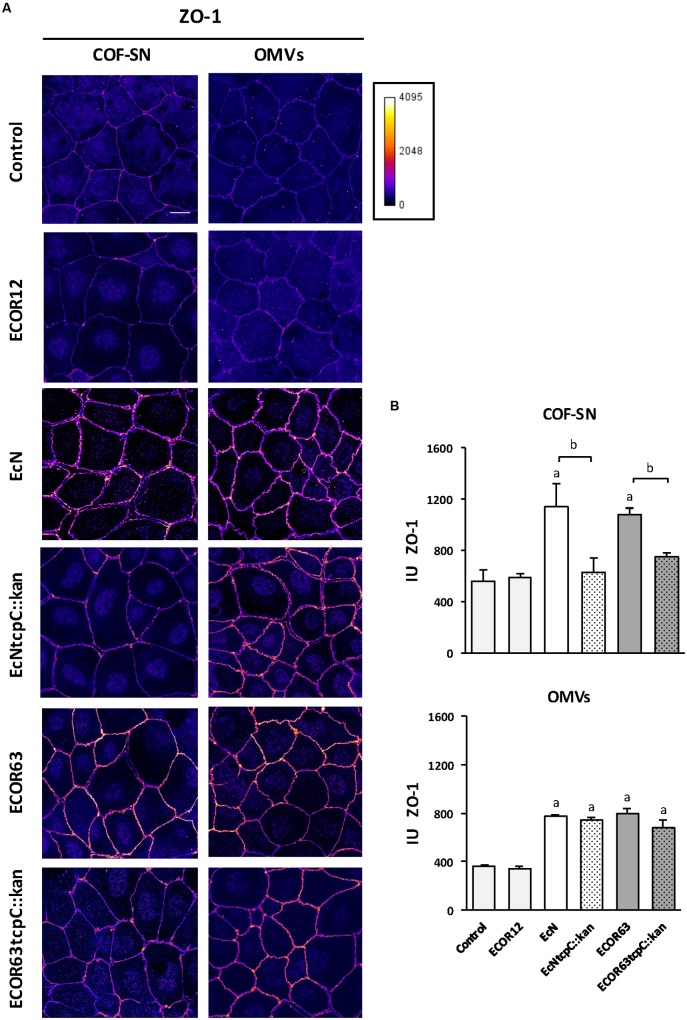
**Immunofluorescence staining for ZO-1 in Caco-2 cells treated for 24 h with COF-SN or OMVs from the indicated bacterial strains.** **(A)** Immunostaining of ZO-1 was carried out in Caco-2 cells at 5 days after seeding. Images are color coded with Fire look-up table and its calibration bar is shown on the right. Scale bar, 20 μm. **(B)** Quantification of the mean fluorescence intensity of ZO-1 labeling in tight junctions. See **Supplementary Figure S2** for details in the tracing and processing of the images. Data are presented as mean ± SEM of relative intensity units (IU) (*n* = 5 independent biological replicates for COF-SN treated cells and *n* = 3 independent biological replicates for OMVs treated cells). Statistical differences were assessed by the *t-*test. ^a^Significance against untreated control cells (*p* < 0.015); ^b^significance *tcpC* mutant vs. wild-type (*p* ≤ 0.05).

To confirm the impact of TcpC and other soluble and vesicle-secreted factors on TJ, we carried out immunofluorescence staining, followed by confocal laser scanning microscopy of ZO-1 and claudin-2. Occludin was analyzed in parallel as a control of a non-regulated protein. This analysis was performed in Caco-2 monolayers challenged for 24 h with COF-SN or OMVs isolated from wild-type EcN and ECOR63 and the derived *tcpC* mutants. Untreated Caco-2 cells and Caco-2 cells incubated with samples collected from ECOR12, which did not exhibit the ability to strengthen the epithelial barrier, were processed in parallel as a control. After 24 h incubation, cells were fixed and stained for the indicated proteins. Representative images are presented in **Figure 5** (for occludin and claudin-2) and **Figure 6** (for ZO-1). As expected, no differences in the fluorescence signal of any of the three proteins were observed in cells treated with ECOR12 samples with respect to the untreated control cells. The results for cells incubated with EcN and ECOR63 samples correlated with mRNA and protein expression data. Consistently, no significant treatment-associated changes were observed for occludin, whereas the claudin-2 signal was diminished in cells treated with either COF-SN or OMVs isolated from EcN and ECOR63, independently of whether they were positive or negative for TcpC (**Figure 5**). Notice that the claudin-2 signal was mainly localized in the cytoplasm. Since our analysis was performed in cells grown for 7 days, this observation is consistent with results from a time course analysis of claudin-2 immunofluorescence staining in Caco-2 cells, reported elsewhere (Fisher et al., 2014). After 3 days of cell seeding, the claudin-2 signal appeared to be diffusely distributed in the cytoplasm, whereas the progressive location of claudin-2 along TJ could be observed later during cell differentiation, such as at 21 days (Fisher et al., 2014). Concerning the upregulated ZO-1 protein, microscopy images showed an increased signal in the cell boundaries of Caco-2 monolayers treated with wild-type EcN or ECOR63 COF-SN, whereas this increase was not apparent in cells incubated with COF-SN collected from cultures of the *tcpC* mutants. In this case, ZO-1 signals were similar to those of control cells (**Figure 6A**). OMVs isolated from EcN and ECOR63 also promoted an increase in the peripheral ZO-1 signal, and this effect was not abolished by the *tcpC* mutation (**Figure 6A**).

The ZO-1 signal was quantified in TJ, as described in the section “Materials and Methods” and illustrated in **Supplementary Figure S2**. The results presented in **Figure 6B** reveal a statistically significant increase in TJ ZO-1 after treatment with COF-SN from EcN and ECOR63, but not after incubation with supernatants from the derived *tcpC* mutants (data collected from five independent biological experiments, mean of total cells analyzed for each experiment = 94 ± 6.16). Quantification analysis also confirmed that the ZO-1 increase triggered by EcN or ECOR63 OMVs was TcpC-independent (data collected from three independent biological experiments, mean of total cells analyzed in each experiment = 104 ± 15.30). Quantification of the occludin signal using the same software ruled out significant changes in the junctional localization of this protein between treated and untreated cells. The analysis of three independent replicates with a mean of 127.26 ± 28.43 cells per experiment yielded arbitrary intensity units around 500–600 for all conditions (not shown).

## Discussion

It is now well-known that gut microbiota is a source of regulatory signals that influence the maturation and function of the digestive and immune systems. In this context, the intestinal tract requires barrier and regulatory mechanisms to control reciprocal interactions between commensal bacteria, the epithelium and the mucosal immune system that prevent aberrant responses and preserve homeostasis. Conditions that compromise the integrity of the epithelial barrier are the basis of a wide range of illnesses, with special impact on inflammatory bowel diseases. As certain commensal bacteria modulate the integrity of the epithelial barrier, potential clinical applications of gut microbes are being explored to reduce increased intestinal permeability and improve the clinical status of such gastrointestinal diseases. Besides anti-inflammatory effects, upregulation and redistribution of TJ proteins are among the multiple mechanisms used by these microbes to improve the epithelial barrier function. The probiotic EcN is a good intestinal colonizer that has been licensed for use in human medicine to treat several disorders of the gastrointestinal tract. Its therapeutic efficacy in the remission of ulcerative colitis has been proved through clinical trials (Kruis et al., 2004; reviewed by Scaldaferri et al., 2016). In addition, the effectiveness of this probiotic in the amelioration of induced experimental colitis in mice is well-documented (Grabig et al., 2006; Ukena et al., 2007; Garrido-Mesa et al., 2011; Olier et al., 2012; Souza et al., 2016). The ability of EcN to decrease intestinal permeability and cure leaky gut may be attributed, at least in part, to its ability to strength TJ. *In vivo* upregulation of ZO-1 by EcN suspensions was evidenced in both healthy and dextran sodium sulfate-treated mice (Ukena et al., 2007), whereas *in vitro* studies showed upregulation of ZO-2 (Zyrek et al., 2007) or claudin-14 (Hering et al., 2014), depending on the epithelial cell line used.

In the gut, communication between microbiota and the host mainly relies on secreted factors that can go through the mucus layer and reach the epithelium. Thus, membrane vesicles released by commensal strains are emerging as key players in signaling processes in the intestinal mucosa. We have recently shown that EcN OMVs, as well as vesicles produced by other commensal *E. coli* strains, are internalized by epithelial cells through clathrin-mediated endocytosis (Cañas et al., 2016) and mediate signaling events to the immune system through the intestinal epithelial barrier (Fábrega et al., 2016). Here we aim to analyze the impact of EcN OMVs on the integrity of the intestinal epithelial barrier.

In HT-29/B6 monolayers, the TER increase and upregulation of claudin-14 by EcN was attributed to TcpC, a secreted protein present in the culture supernatant. No other regulatory effects on TJ proteins were apparent in this cellular model (Hering et al., 2014). Upon removal of bacteria, culture supernatants contained both OMVs and soluble factors. Thus, we isolated the released vesicles from the putative active factors secreted in a soluble form (COF-SN), and tested both extracellular fractions independently. We extended our study to other *tcpC*-positive *E. coli* strains from the ECOR reference collection, and confirmed that their culture supernatants stimulated TER in T-84 cell monolayers. Like EcN, these *tcpC*-positive isolates fit into the phylogenetic group B2, which is associated with virulent strains that cause extra-intestinal infections. In fact, TcpC is highly prevalent in Gram-negative pathogens. This protein contains a Toll/IL-1 receptor (TIR)-binding domain, which mediates its interaction with Myd88 and inhibits toll-like receptor signaling pathways. By this mechanism, TcpC impairs innate immunity, causing inflammation and tissue damage (Cirl et al., 2008; Yadav et al., 2010). However, in spite of its virulent nature, TcpC have a positive impact on the epithelial barrier (Hering et al., 2014). TcpC, like the immunomodulin colibactin, are among the virulence factors encoded in the EcN genome that contribute to its probiotic activity. Colibactin is a non-ribosomal peptide-polyketide synthesized by enzymes encoded in the *pks* island, which displays both genotoxic and anti-inflammatory effects. Mutations that abolish colibactin synthesis greatly reduce the beneficial activity of EcN in the modulation of cytokine expression and amelioration of experimental colitis in mice (Olier et al., 2012). The prevalence of extraintestinal virulence determinants in commensal *E. coli* B2 isolates has been explained on the basis that these determinants are in fact survival factors that can increase the fitness of the strains within the normal gut environment (Le Gall et al., 2007).

Our results showed that the strengthening activity of EcN and ECOR63 (the selected *tcpC*-positive strain lacking cytotoxic activity) does not exclusively depend on TcpC. Several facts point to the contribution of other bacterial effectors. First, partition of the culture supernatants revealed that both OMVs and soluble factors (COF-SN) collected from these strains increased the TER of T-84 cell monolayers upon 24 h incubation. Second, TcpC deficiency did not alter the OMV-stimulatory activity, and only diminished the effect of COF-SN collected from EcN. Overall, these results show for the first time the ability of secreted microbiota vesicles to modulate the integrity of the epithelial barrier, and also indicate that TcpC is not exported through OMVs. In addition, they reveal that these strains, especially ECOR63, secrete soluble active factors other than TcpC that have a great impact on the epithelial barrier.

Quantitative reverse transcription PCR analyses were undertaken to evaluate the capacity of these soluble and vesicular-secreted fractions to regulate the expression of selected TJ proteins. Neither OMVs nor COF-SN from the two strains triggered significant changes in the mRNA levels of ZO-2, occludin or claudin-1. Regarding ZO-2, studies performed in the same cellular models used here (T-84 and Caco-2 cells) revealed upregulation of this protein upon incubation with EcN suspensions (Zyrek et al., 2007). As we stimulated the epithelial cell monolayers with secreted EcN fractions, we may speculate that EcN-mediated upregulation of ZO-2 depends on bacteria-associated factors. Concerning the other genes analyzed in this study, our results showed upregulation of claudin-14 and ZO-1, and downregulation of claudin-2. Interestingly, all these effects were mediated either by OMVs or COF-SN collected from the two strains.

Regarding claudin-14, upregulation of this protein by COF-SN samples was in agreement with the literature on EcN supernatants, as this effect mainly relies on TcpC (Hering et al., 2014). Nevertheless, in ECOR63, TcpC deficiency did not result in a significant reduction of claudin-14 mRNA levels, at least in the T-84 cellular model. This confirms the presence of other active soluble factors in the COF-SN samples from ECOR63. Moreover, OMVs isolated from both EcN and ECOR63 strains also triggered upregulation of claudin-14 in a TcpC-independent manner.

In the case of ZO-1, our results indicate that the *in vivo* upregulation of this protein by EcN (Ukena et al., 2007) may be attributed to TcpC and released OMVs. Here, expression of this protein was analyzed at both mRNA and protein levels. In all conditions tested, ZO-1 protein quantified by Western blot correlated well with the corresponding mRNA levels, which points to transcriptional regulation of the gene. As expected, upregulation of ZO-1 by bacterial OMVs did not depend on TcpC. Regarding the activating activity of COF-SN samples, the results differed depending on the strain. In EcN, upregulation of ZO-1 by COF-SN depends on TcpC exclusively, but in ECOR63 other soluble secreted factors existing in the corresponding sample could contribute to modulating ZO-1 expression. Notice that ZO-1 mRNA levels in T-84 cells treated with COF-SN from ECOR63 *tcpC::kan* were still significantly higher than those of untreated controls. Immunofluorescence staining followed by confocal laser scanning microscopy evidenced enhanced ZO-1 staining in the cell boundaries of monolayers treated with EcN or ECOR63 samples, but not in cells incubated with ECOR12 samples. In stimulated Caco-2 monolayers, enhanced expression of ZO-1 correlates with an increased TJ-associated ZO-1 signal, except for cells treated with COF-SN collected from the ECOR63 *tcp* mutant. In this case, the increase in TJ-associated ZO-1 signal was not statistically significant.

In addition to TJ proteins that are known to be regulated by EcN, in this study we included the leaky protein claudin-2, which plays an opposing role to other TJ proteins. This is a pore-forming protein that facilitates cation and water secretion by epithelial cells. Stimuli that raise claudin-2 levels result in increased barrier permeability (Luetting et al., 2015). Increased expression of claudin-2 has been reported in the gut epithelia of patients with ulcerative colitis and Crohn’s disease (Hering et al., 2012; Landy et al., 2016). Pathogens like Salmonella increase claudin-2 expression to facilitate bacterial invasion (Zhang et al., 2013). Microbial toxins, such as cholera toxin or Staphylococcal enterotoxin B, also promote upregulation of claudin-2, and thus compromise the intestinal epithelial barrier (Liu et al., 2013). In contrast, downregulation of claudin-2 is part of the mechanism used by some probiotic strains to enhance the intestinal barrier function (Ewaschuk et al., 2008). Our study shows for the first time the capacity of EcN to decrease claudin-2 expression. By means of gene and protein expression analyses, we provide evidence that downregulation of claudin-2 by EcN is mediated by released OMVs as well as by soluble factors, other than TcpC. In addition to the known effects of EcN on expression of the sealing TJ proteins ZO-1 and claudin-14, downregulation of claudin-2 can contribute to the efficacy of this probiotic in the treatment of intestinal infections and inflammatory diseases. Secreted OMVs and microbial factors from ECOR63, which belongs to the phylogenetic group B2 like EcN, also decrease claudin-2 expression. However, commensal ECOR12 (phylogenetic group A), which does not reinforce the TER of the epithelial barrier, does not modulate claudin-2 expression.

## Conclusion

This study addresses the impact of bacterial secreted factors on the expression of TJ proteins that are known to be regulated by EcN, as well as other proteins described here for the first time, like claudin-2. To the best of our knowledge, this is the first study providing evidence that OMVs from certain commensal *E. coli* strains, specifically EcN and ECOR63, increase epithelial barrier function through upregulation of ZO-1 and claudin-14, and downregulation of the leaky protein claudin-2. These regulatory effects are not mediated by TcpC. We have previously reported that OMVs released by the probiotic EcN induce IL-22 expression in colonic explants (Fábrega et al., 2016). This cytokine, mainly expressed by immune cells, targets epithelial cells and reinforces the intestinal barrier, thus limiting the access of microbial compounds and allergens to the systemic circulation. Thus, our studies show that OMVs contribute to reinforcing the epithelial barrier integrity directly through transcriptional regulation of TJ proteins and indirectly through regulation of IL-22.

Besides OMVs, the soluble secreted TcpC protein also contributes to the upregulation effect of ZO-1 and claudin-14. In contrast, this protein has no effect on the transcriptional regulation of claudin-2. Thus, these microbiota strains release other bioactive factors that contribute to the reinforcement of the epithelial barrier.

## Author Contributions

LB, RG, and JB conceived of the study with the participation of MB and C-SA in experimental design. LB and JB wrote the manuscript. LB and RG supervised the work. RG, MB, and C-SA carried out data interpretation and statistical analysis. C-SA performed the experimental work. MB participated in confocal microscopy experiments and carried out image analysis. All authors revised, read and approved the final manuscript.

## Conflict of Interest Statement

The authors declare that the research was conducted in the absence of any commercial or financial relationships that could be construed as a potential conflict of interest.

## Footnotes

1 http://www.ncbi.nlm.nih.gov

2 http://www.shigatox.net/new/reference-strains/ecor.html

## Supplementary Material

**FIGURE S1 | PCR confirmation of gene disruption in EcN *tcpC::kan* and ECOR63 *tcpC::kan* mutants.** PCR amplification of *tcpC* sequences with primers *FW-tcpC* (TCGCATGGAAACAGCACCT) and *RV-tcpC* (CTTCTCCTGTATGCTAT TTCAGC) in the indicated strains. The size of the amplified products is indicated. The 1.6 Kb-increase in the PCR product from knockout mutants corresponds to the kanamycin cassette inserted inside *tcpC* gene. The size marker was lambda HindIII.

**FIGURE S2 | Image analysis of tight junction stained with ZO-1.** **(A)** Inset of an original image. **(B)** Same inset after processing by median filter, subtract background and tracing using the Tubeness plugin. **(C)** Regions of interest (ROIs, green) obtained after binarizing **(B)** by applying Li’s Minimum Cross Entropy thresholding method. **(D)** Overlap of ROIs in **(C)** on the original image **(A)**. Images are color coded with Fire look-up table. Scale bar, 20 μm.

## Abbreviations

CF-SN: cell-free supernatant
COF-SN: cell-OMV-free supernatant
DMEM: Dulbecco’s modified Eagle medium
EcN: *Escherichia coli* Nissle 1917
FCS: fetal calf serum
LB: Luria-Bertani broth
OMVs: outer membrane vesicles
PAGE: polyacrylamide gel electrophoresis
PBS: phosphate buffer saline
RT-qPCR: quantitative reverse transcription PCR
SDS: sodium dodecyl sulfate
TER: transepithelial electrical resistance
TJ: tight junctions
ZO: zonula occludens
