# Supplementary figures and images for: Outer Membrane Vesicles and Soluble Factors Released by Probiotic Escherichia coli Nissle 1917 and Commensal ECOR63 Enhance Barrier Function by Regulating Expression of Tight Junction Proteins in Intestinal Epithelial Cells
Source: Front Microbiol. 2016 Dec 15;7:1981. doi: 10.3389/fmicb.2016.01981 (PMC5156689; doi:10.3389/fmicb.2016.01981)

Supplementary Figure 1

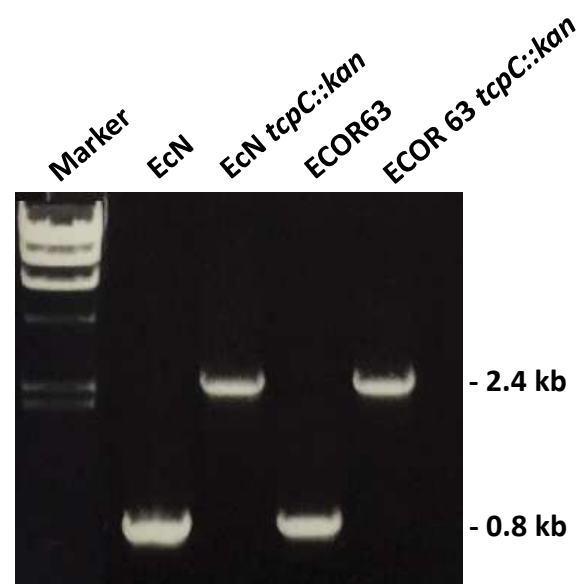

Supplement: Supplementary file 1 [file Image_1.PDF]

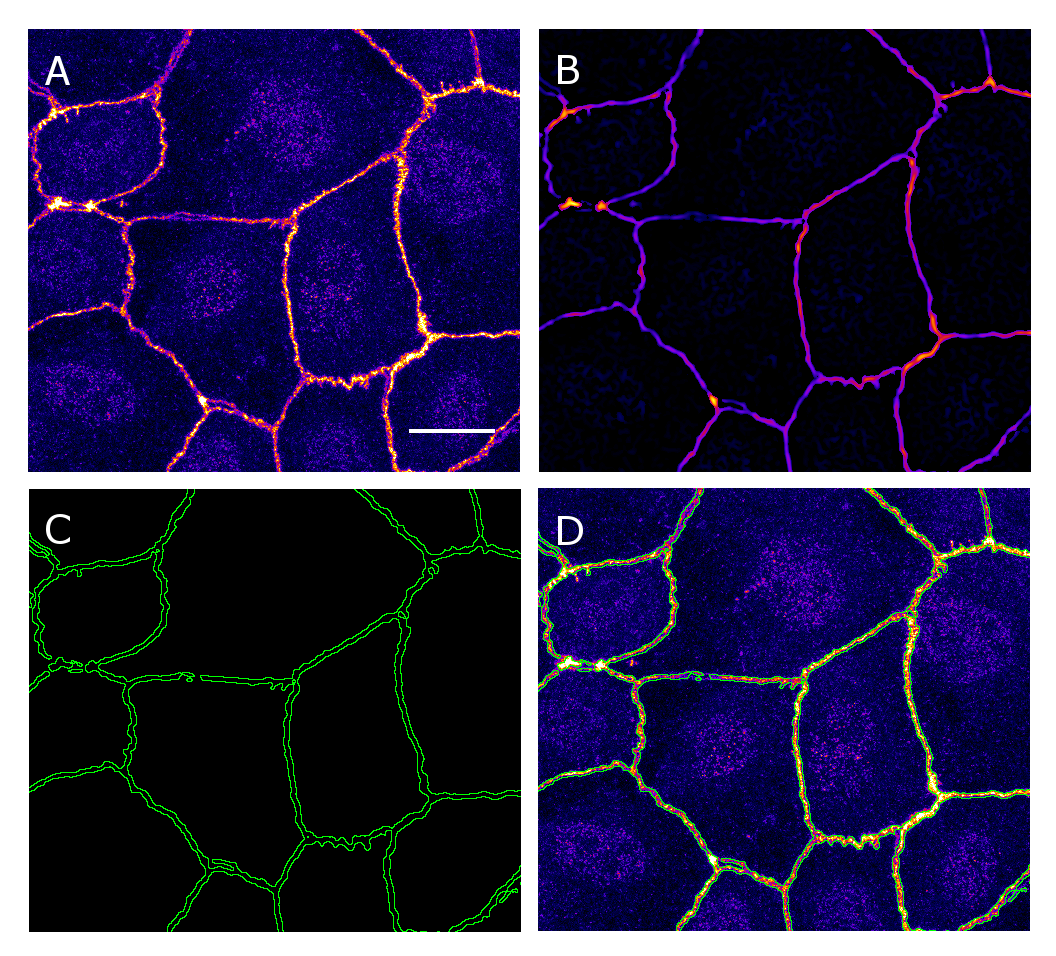

Supplement: Supplementary file 2 [file Image_2.TIF]
